# The Story of the Insane from Year to Year

**Published:** 1902-07-05

**Authors:** 


### THE STORY OF THE INSANE FROM YEAR TO YEAR.

### Fourteenth Annual Series.

### THE GARTLOCK ASYLUM, GLASGOW.

The insane population of this asylum increased during the year from 451 to 479. There were 156 first admissions, 55 readmissions, 77 recoveries, 59 discharged relieved, and 45 deaths. The average number resident during the year was 469, and the total number of persons under treatment was 653. The recoveries were 36·5 per cent. of the admissions, and the deaths were 9 6 per cent. of the average number resident. Of the year's deaths 21 were caused by cerebral and spinal diseases, 8 by consumption, 5 by other lung diseases, and 1 by enteritis. Of the admissions 5 owed their insanity to moral causes, and the chief physical causes were intemperance in drink which accounts for 33, old age for 13, and hereditary influence for 49. In addition to the 8 cases of consumption, it is probable, says Dr. Oswald, that tuberculosis was a contributory cause in some of the other 18 cases in which tubercle was found on post-mortem examination. A system of isolation of consumptive patients is in force here. A new home to accommodate a large number of nurses has been opened, and it is hoped that this and other advantages conferred on the staff will induce them to remain in the service. Dr. Oswald remarks that "abundance of fresh air, exercise and sunshine, are often the best prescriptions we can give our patients, but such a prescription cannot be best taken in a climate where 42 inches of rain falls and where only 160 days in the year are dry." This makes one think that Gartloch is not altogether a desirable place in which to live, and there may be something in Dr. Oswald's wish that the Glasgow Corporation should give him more sunshine that he may reduce the lunacy assessment; but is not he aware that England has more sunshine than Scotland and also more lunacy? This is only the third annual report of the asylum and yet it will be full in two or three years, and the committee are advised, and rightly so, to take into consideration what steps should be taken in the matter. Delay is expensive in these affairs. The committee record their continued confidence in the whole official staff, and especially Dr. Oswald. The maintenance rate is 10s. per head per week.

### THE RETREAT, YORK.

THIS institution for the insane contained 164 patients at the end of the year, being only 2 more than it had in residence at the beginning of it. There were 27 first admissions, 10 readmissions, 14 recoveries, 8 discharged relieved, and 8 deaths. The average number resident during the year was 164, and the total number under treatment was 197. Calculated on the admissions, the recoveries were 37·84 per cent., and the deaths were 4·87 on the average number resident. Three of the deaths were from general paralysis of the insane, and 1 was from consumption. Intemperance in drink accounted for the insanity in 2 of the admissions, and hereditary influence in 16. This hospital has always been noted for the amount of charitable work done by it, and this year is no exception. While the average weekly cost per patient was £2 5s. 10d., it is a most pleasing fact to note that 36 were paying 12s. a week, 21 were paying between 12s. and 21s., and 51 between 21s. and 42s. These figures show that 108 patients out of a total of 164 have paid less than the actual cost, and some of them very much less. To encourage outdoor occupation among the patients, it is the practice in this asylum to pay those employed in the grounds a sum of 3d. an hour. It is a good plan for promoting the health and contentment of the patients, but it is not surprising to learn that it is a doubtful expedient from a business point of view. Dr. Pierce does not forget his staff, and has done something to reduce the hours spent on duty by the attendants and nurses. Probationers are received, and this new movement seems to answer. Dr. Pierce adds that "there is opening out a new sphere of work for educated women, which in the future will have an important influence on the welfare of the insane." Twenty-two nurses left of their own wish during the year, apparently to get married, for it is stated that matrimony had "dealt hardly with us." We hope it has dealt mildly with the couples themselves.

### DUNDEE ROYAL ASYLUM.

AT the beginning of the year there were 402 patients in residence, and at the end of the year there were 412. The admissions were 107, readmissions 18, recoveries 43, discharged relieved 32, died 38. The total number under treatment was 526, and the average number resident was 410. The recoveries stand at 34 40 per cent. of the admissions, and the deaths at 9·26 per cent. on the average number resident. Of the 38 deaths, 20 were caused by cerebral and spinal diseases, 5 by consumption, and 1 by pneumonia. Moral causes account for 33 of the year's admissions, drink for 32, hereditary influences for 10, and previous attacks for 33. There are 110 private patients in this asylum, and the payments range from £25 to £180 per annum.

### RAINHILL ASYLUM, LANCASHIRE.

THIS asylum contained 2,088 patients on January 1st and 2,092 on the last day of December. There were 397 first admissions, 36 readmissions, 147 recoveries, 14 discharged relieved, and 255 deaths. The total number under treatment was 2,517, and the average number resident was 2,094. The recoveries were 33·94 per cent. of the admissions, and the deaths were 12·17 per cent. of the average number resident. Of the deaths 82 were caused by cerebral and spinal diseases, 70 by consumption, 37 by other lung diseases, 1 by enteric fever, and 5 by colitis. Of the admissions 61 owed the insanity to moral causes, 186 to intemperance in drink, 53 to hereditary influences, 59 to previous attacks, and 15 to congenital defect. The above figures show that 239 of the year's admissions were caused by heredity and drink; and, as both of these are preventible, it ought to be recognised that legislation of some kind is called for. The deaths from tubercular disease were 27·45 per cent. of the total number of deaths. Dr. Wiglesworth points to the overcrowding of the asylum as one cause, and to our system of asylum window construction as another. As to the latter he is inclined to regard it "as an important factor in the high tubercular death-rate of asylums." We are tolerably sure Dr. Wiglesworth is right in his opinion, and we hope that he will impress his views on the Commissioners in Lunacy and induce them to change their requirements in this respect. But it is part of a system which needs revising, as some of the latest-built asylums demonstrate, and no part more urgently than the lack of efficient cross-ventilation of the dormitories. Dr. Wiglesworth is in favour of detached hospital buildings for phthisical patients, and in large asylums like Rainhill there ought to be no difficulty in organising these hospitals. The weekly maintenance rate was 8s. 11¾d. per head.

